# Precision Feeding in Ecological Pig-Raising Systems with Maize Silage

**DOI:** 10.3390/ani12111446

**Published:** 2022-06-03

**Authors:** Yun Lyu, Jing Li, Ruixing Hou, Yitao Zhang, Sheng Hang, Wanxue Zhu, He Zhu, Zhu Ouyang

**Affiliations:** 1 College of Grassland Science and Technology, China Agricultural University, Beijing 100193, China; lvyun@cau.edu.cn; 2 University of Chinese Academy of Sciences, Beijing 100049, China; jingli@igsnrr.ac.cn (J.L.); hourx@igsnrr.ac.cn (R.H.); zhangyt@igsnrr.ac.cn (Y.Z.); hang_some@126.com (S.H.); zhuwx.16b@igsnrr.ac.cn (W.Z.); 3 Yucheng Comprehensive Experiment Station, Key Laboratory of Ecosystem Network Observation and Modeling, Institute of Geographic Sciences and Natural Resources Research, Chinese Academy of Sciences, Beijing 100101, China; 4 Yellow River Delta Modern Agricultural Engineering Laboratory, Institute of Geographic Sciences and Natural Resources Research, Chinese Academy of Sciences, Beijing 100101, China; 5 Key Laboratory of Regional Sustainable Development and Modeling, Institute of Geographic Sciences and Natural Resources Research, Chinese Academy of Sciences, Beijing 100101, China

**Keywords:** ecological pig-raising system, maize silage, big data, precision feeding, pig growth modeling

## Abstract

**Simple Summary:**

Generally, ecological farming is regarded as an environmentally friendly production process with high costs. The plant fiber source (e.g., maize silage) has become a common practice to reduce the cost during the fattening period to gain more profit, which means a precision feed component is needed in ecological pig-raising systems (EPRS) for achieving the balance between environment and economic profit. This manuscript provided a suitable and easy-operated ratio of sun-dried maize silage added to the feed. Meanwhile, we tried to reveal the trends of the pig growth, environmental impacts, and economic profits with the sun-dried maize silage percentage and raising period increasing. Results showed that the best balance point between environmental impact and economic performance was 20% sun-dried maize silage added to the feed with a 360-day raising period.

**Abstract:**

Ecological pig-raising systems (EPRSs) differ from conventional breeding systems, focusing more on environmental consequences, human health, and food safety during production processes. Thus productions from EPRSs have undergone significant development in China. Thus far, adding plant fiber sources (e.g., sweet potato leaves, maize or wheat straw, potato, alfalfa, and vinasse) to feed has become a common practice to reduce the cost during the fattening period. Under such a context, it is necessary to choose the precision EPRS diet components and fattening period with low environmental consequences and high economic benefits. This study set up a database via pig growth models to predict environmental and economic performance based on two trials with 0%, 10%, 40%, 60%, and 80% maize silage (dry weight) added to the feed. A continuous curve about plant fiber concentration was built through the generated database. Our results showed that, with increased plant fiber concentration, the environmental performance of the EPRSs exhibited an “increase-decrease-increase” trend, and the economic performance firstly increased and then decreased. The best maize silage added percentages of emergy yield ratio (EYR), environmental loading ratio (ELR), unit emergy value (UEV), and emergy sustainability index (ESI), and the economic profits were 19.0%, 34.3%, 24.6%, 19.9%, and 18.0%, respectively. Besides, the 19.9% sun-dried maize silage added to the feed with a 360-day raising period had the best balance for environmental impact and economic performance. At the balance point, the performances of EYR, ELR, UEV, ESI, and the economic profit were only 0.04%, 3.0%, 0.8%, 0.0%, and 0.1%, respectively, lower than their maximum values. Therefore, we recommended the feed added 20% sun-dried maize silage is suitable for practical pig raising systems.

## 1. Introduction

In China, meat is a large part of the dietary structure, especially pork, reaching 64.1% of the total meat production during the last five years [1]. Therefore, much demand for pork stimulated the growth of large-scale and intensive pig production [2,3]. However, the vast amount of waste discharged from such raising systems can cause serious environmental problems [4,5,6]. Meanwhile, with improvements in living standards, the increasing population pays more attention to food quality. Under such conditions, production according to China Green Food certification has received more attention [7]. Green foods are agricultural products with China Green Food certification. Generally, such green foods mainly come from ecological systems; the ecological raising systems allow the proper application of modern science and technology within the scope of ecological and economic principles [8]. Ecological pig-raising systems (EPRSs) use some unique methods to gain better environmental performance. The above approaches include applying microbiological agents instead of chemical agents to disinfect and control odors, using slatted floors to reduce water consumption, and without heavy-metal or synthetic additives in the diets [9]. To some degree, ecological raising systems can deal with both environmental pressure and economic profit [10].

EPRS system aims to benefit the environment during the pig raising process. The EPRS adopts modern sciences and technologies and system engineering methods following ecological and economic principles for more economic benefits and to protect natural resources. For example, the application of the biological agents instead of chemical agents, the application of the slatted floor to reduce the water wasting, and no use of heavy-metal or synthetic additives in the diets. EPRSs provide a balance point between the environment and profit. However, a large number of inputs and long raising period limit improvements to EPRSs [11]. Generally, EPRSs reduce feed costs and sell pigs at light weights to avoid these two disadvantages [12]. As a kind of monogastric animal, Pigs can digest some forage crops [13,14,15]. During the fattening period, some plant fiber (crop byproducts, forage, etc.) is added to pig feed for reducing the cost [16]. The concentration of plant fiber added to the feed affects the pigs’ growth rate. Generally, the percentage of plant fiber added was according to experience. Nearly no one knew how the environmental impact and economic profit would change with increased maize silage percentage. Therefore, feeding the appropriate concentration of plant fiber and adopting a convenient raising period can benefit the environment and economy more [17,18].

Previous studies have analyzed the performance of different pig production systems [18,19]. However, these studies just focused on comparing two existing systems [20,21], and they did not provide quantitative indices to improve system performance. Most data in current studies for emergy analysis came from survey questionnaires or statistical yearbooks instead of a direct data collection. Meanwhile, current studies focused on the pigs’ biological mechanism or production performance when plant fiber was added to the feed, without considering the entire raising system [22,23,24]. Furthermore, few integrated the growth models and emergy analysis to provide quantitative indices for improving an existing system, although limited studies developed quantitative indices using other methods [25,26,27]. Consequently, it is essential to develop growth models by directly collecting data, and assess the performance of the model outputs with different plant fiber concentrations and fattening periods in terms of the environment and economy. In this study, following the dominant cropping patterns in the North China Plain, i.e., winter wheat–summer maize, we took maize silage as the plant fiber added to the feed. The objectives of this study were to (1) provide a new method to output quantification results considering both environmental performances and economic profits, (2) reveal the environmental performance and profit trends with an increased concentration of plant fiber and extended fattening period, and (3) offer a balance point, which could guide the factual pig raising based on the model simulation.

## 2. Materials and Methods

### 2.1. Study Site

**Figure 1:**
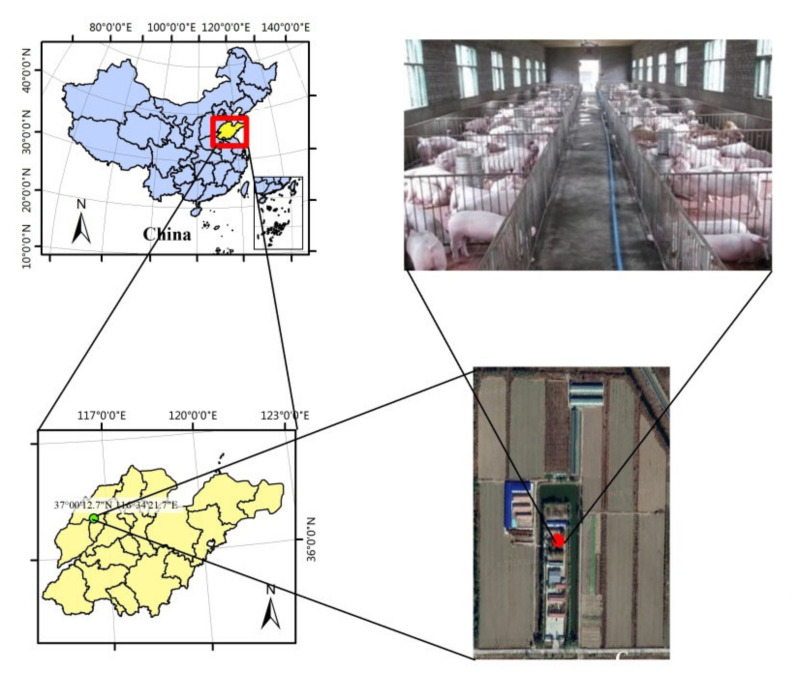
Location of the study area and the overview of ecological farm structure.

This study developed five EPRSs via simulation at the Beiqiu Farm (37°00′ N, 116°34′ E), located in Dezhou City, Shandong Province. Beiqiu Farm belongs to the Yucheng Comprehensive Experiment Station, Chinese Academy of Science, and aims to build a typical ecological family farm in the alluvial plain of the Yellow River (Figure 1). Beiqiu Farm could be a typical representation of a pig raising farm in the North China Plain [28]. The study area has a warm, temperate, semi-humid monsoon climate. The mean annual temperature and frost-free period are 13.1 °C and 200 d, respectively. In 2014–2016, the average yearly precipitation and wind speed were 451 mm and 2.413 m/s, respectively; the annual average solar radiation was 4936 MJ/m^2^, including 2640 h of sunshine annually.

The Beiqiu farm covers 15.3 ha with about 10 ha of planting area and 1.5 ha of ecological pig production area. All feed except soybean meal consumed in the ecological raising system was obtained from the planting area. The feed at Beiqiu Farm is mainly silage, maize, soybean, and wheat bran, without any chemical additives. Vaccines and medicines are not used unless necessary to cure existing diseases. During the raising period, microbiological additives are used for disinfection.

### 2.2. Experimental Design

According to previous studies, the best production performance point was below 10% roughage addition [29,30,31,32]. We assumed the best production performance point might gain better environmental performance. With the limit of trails, we set a 10% addition level instead of a 20% level, aiming to achieve a result close to the actual effect. This study involved two trials. The first trial lasted from 27 July 2017 to 11 January 2018, with 0% and 40% maize silage (dry) added to the feed. The second trial was an extended experiment with 10%, 60%, and 80% maize silage (dry) added to the feed, from 6 September 2018 to 6 January 2019. The maize silage was harvested at the late milk stage. Then, the straw was harvested and packed by a silage baler. When the maize silage was fermented well, the fodder used to feed pigs would be dried by the sun and stored in the barn. All components were mixed with a grinder with a 20-mesh sieve during the feed preparation. In this study, the crude protein content of the maize silage (dry weight) was 10.54 ± 2.03%. The first trial raised 16 crossed pigs, and the second trial raised 15 crossed pigs. Every 4 or 5 crossbred Duroc × (Landrace × Northeastern Indigenous) pigs of both sexes were fed in 20 m^2^ pens.

All pens had concrete slatted floors and were cleaned by a manure scraper under the floor to reduce water consumption. In summer, the pig house used two axial flow fans and wet curtains to maintain the indoor temperature below 30 °C. In winter, when the outdoor temperature was below 0 °C, all windows and doors of the pig house were closed, with no other heating methods. Every day 125 mL of a microbiological agent (ETS Gold Liquid Enzyme, ETS Biotechnology Development Co. Ltd., Tianjin, China) was diluted in 20 L of water and sprayed in the house for daily disinfection and odor control. Beiqiu Farm raises two batches of pigs every year. All piglets are bought from the market. Then, the piglets are raised with the ecological diet (only containing maize grain, soybean meal, and wheat bran meal). The composition of the feed of all EPRSs is listed in Table 1.

### 2.3. Data Collection

In this study, the primary data were the pig weights and the amount of EPRS inputs (water, electricity, feed, medicine, microbiological materials, etc.). Pigs were weighed by an electronic cage scale (Lilang XK3190, Changzhouliliang Electronics Co., Ltd. Changzhou, China) at each growing phase. The amount of water and electricity consumed during the entire raising period was recorded by meters. The feed added every day was recorded as the amount of feed consumed. The amount of feed recorded was measured per pen. Additionally, the feed weight consumed per pig was the recorded data divided by the pig number per pen. The mean values of feed weight considered individual differences were suitable to use in the emergy analysis. Additionally, statistical indicators such as the standard deviation and coefficient of variation were not used for the emery analysis. Other economic data (building materials, equipment, sold price, etc.) were gained from the farm’s account books.

### 2.4. Pig Growth Modeling

A biological growth model reveals the general rules of development. Such models are widely used to predict future production performance in the commercial breeding field [26]. Especially when the experimental conditions are limited, the pig growth modeling provides a way to gain vast amounts of data without setting too many trials [33,34]. Many biological growth models have been developed to describe pig growth and reveal related rules, such as the Logistic, Gompertz, Brody, and Bertalanffy models [35,36,37,38]. The formula of each model is listed below (Equations (1)–(4)). In this case, we calculated each pig growth model using the data collected from experiments and chose the best fitting model to construct the future database.

(1) $$\mathrm{Logistic}:\;W_{t}=a/\left(1+b\times e^{-c\times t}\right)$$

(2) $$\mathrm{Gompertzy}:\;W_{t}=a\times e^{-e^{\left(b-c\times t\right)}}$$

(3) $$\mathrm{Brody}:\;W_{t}=a\times (1-b\times e^{-c\times t}+d)$$

(4) $$\mathrm{Bertalanffy}:\;W_{t}=a/{\left(1+b\times e^{-c\times t}\right)}^{3}+d$$

where W*_t_* is the live weight of the pig at a specific time and *t* is raising time in days.

For pig raising systems, the general raising periods are one or two batches a year. Very few pig raising systems feed pigs for more than a year. A short break between two batches is needed to disinfect the pens and fix some facilities. Thus, six time spans (60, 120, 180, 240, 300, and 360 raising days) were set to reveal the rules of the environmental performance with different concentrations of maize silage added and different raising periods. The soybean meal concentration is 15% in all trials, as soybean meal was the primary source of protein in the diet. It is unclear if the growth models would still fit when the soybean meal concentration decreased, so we set 80% (the percentage of maize silage added to the feed) as the x-axis’s upper limit to ensure the model’s matching degree. Then, the balance point of the suitable diet component and raising period was calculated. In the Discussion section, we predict the changing trend when the percentage of maize silage is extended to 100%.

The systematic deviation of cumulative feed consumption calculated by the model was smaller than that of daily feed consumption. Then, the cumulative feed consumption (CFI) was calculated based on the allometric growth model [39,40]. As our experimental data were not collected from the time the pigs were born, a constant coefficient was used to reflect the cumulative feed consumption from the pigs’ birth to the start of the experiment.

(5) $$\mathrm{CFI}=a\times W_{t}{}^{b}-c$$

where CFI is the cumulative feed intake; W*_t_* is the live weight of the pig at a specific time; *a* is the regularization constant; *b* is the scaling exponent; *c* is feed consumption before pigs taken in the trails.

All model fitting and calculations were carried out by MATLAB R2015b, 1stOpt 1.5 Pro, and Microsoft Excel 2010.

### 2.5. Emergy Analysis

**Figure 2:**
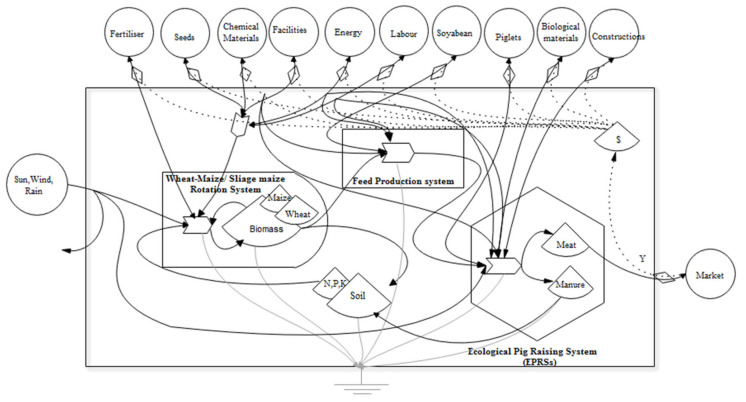
Aggregated system diagrams of EPRSs.

Emergy analysis is a systematic analysis approach that transforms different units of materials and energy and economic data into one standard unit—the solar emjoules (seJ). The unique energy systems language (ESL) can reveal the internal relationships among different parts of an entire system [41,42]. Good evaluation indices derived from the emergy analysis could reflect one system’s integrated performance (Figure 2) [43,44]. The unit emergy value (UEV) reflects the emergy efficiency of the yield and key transformed parameters [45,46]. Emergy yield ratio (EYR) shows the utilization efficiency of emergy invested [45]. The environmental loading ratio (ELR) shows the pressure of the whole system on the environment [43]. The emergy sustainability index (ESI) shows one system’s sustainability degree [43]. These four indexes could provide an integrated evaluation of a system. These advantages make it easier to compare different systems. In this study, UEV, EYR, ELR, and ESI were used to evaluate the various systems (Table 2).

As the two trials lasted from 2017 to 2019, we chose the UEV of the Chinese yuan (¥) in 2018 as the conversion coefficient. There was a near-linear correlation between actual gross domestic product (GDP) and total emergy inputs. The UEV of the Chinese yuan (¥) in 2018 was calculated based on the UEV (7.27 × 1011 seJ/¥ with 9.26 × 1024 seJ/year baseline) calculated by Yang et al. (2010) in 2005 [47]. The GDP deflator from 2005 to 2018 was 3.03 [48]. The UEV of the Chinese yuan (¥) in 2018 was 3.11 × 1011 seJ/¥ (12.0 × 1024 seJ/year baseline). In this study, the global emergy baseline was 12.00 × 1024 seJ/year. All UEVs from other baselines were converted to the same baseline by multiplying by a coefficient.

### 2.6. Economic Analysis

Economic benefits can stimulate the formation and development of a system. For most farmers, the dominant aim is to earn more profit. If a new environmentally friendly technology or method increases profit, it could be considered in practical applications. In this study, we chose profit as the economic index (profit per live pig body weight) to reflect the performance of a system in terms of economic aspects. Profit can reflect the cost and price of production indirectly. The economic analysis was calculated based on the inputs and outputs of systems.

## 3. Results

### 3.1. Pig Growth Modeling

Based on the raw data, the models were calculated by 1stOpt software with nonlinear fitting. The raw data of the experiments are listed in Table 2, and the calculated models are listed in Table 3. Comparing the results of generated models, the maximum body weights of groups A, B, C, D, and E with corresponding values of 188.8 kg, 370.8 kg, 224.6 kg, 153.8 kg, and 148.6 kg, respectively, were obtained from the logistical growth model. Among the maximum weight, group B (10% sun-dried maize silage added) had the greatest potential to reach the heaviest live weight. However, group E (80% sun-dried maize silage added) with the smallest coefficient *a* value (Equation (2)) had the least weight.

In order to reveal the rules about the effects of time span and maize silage concentration on the entire raising system, we chose 60, 120, 180, 240, 300, and 360 days to obtain W*_t_* and CFI data. The time spans reflected the actual pig raising period (1–4 batches a year) based on different raising methods. The data details were the basics in the emergy and economic evaluation (Table 4). The initial weight was 40 kg, as the feed component in these trials stayed fixed when the pig’s live body weight reached 40 kg.

### 3.2. Emergy Analysis

The emergy input and output details can be found in the Supplementary Materials (Table S1). The main emergy indices of the EPRSs are shown in Table 5, which was the basic data used to draw matched curves.

#### 3.2.1. EYR Trend

**Figure 3:**
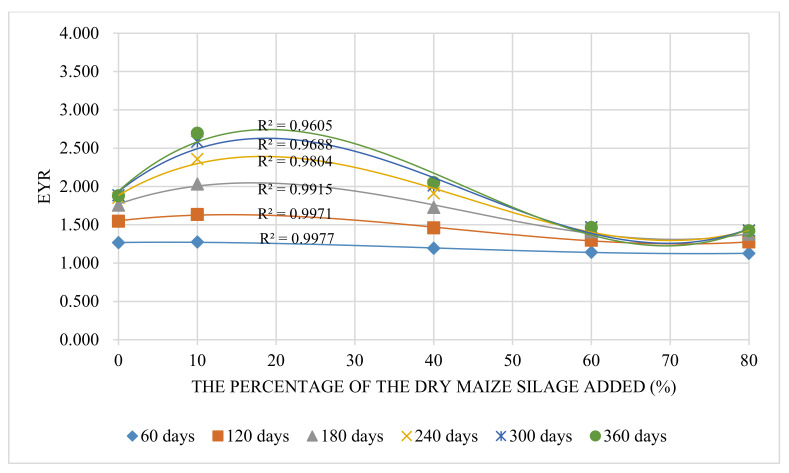
The trend of EYR change with different percentages of maize silage added.

Generally, higher EYR means better production capacity by investing emergy from outside. The EYR results showed that, from 60 to 360 days, the EYR increased with raising growing period (Table 5). The matched curves of maximum EYR and raising period had a linear relationship, suggesting the longer periods boost the increasing of EYR. In this study, 360 days was the upper limit for the EPRS, so the best raising period was 360 days. On the other side, the fitting curves of EYR and the percentage of maize silage were cubic equations (Figure 3), and the two extremes became clearer with the increased raising time. The first extreme EYR values are mainly located in the range of 10–30% of maize silage added, and the minimum EYR values are found in the range of 60–80%. The best raising period was 360 days, the best percentage of maize silage added was 19.0%, and the other extreme point (69.5%) with 360 days was only 34.5% of the maximum EYR value. The above results indicate that 19.0% maize silage added to the fodder with a 360-day raising period achieved the highest EYR value. This mainly depended on the considerable amount of maize silage with less UEV value counteracting the disadvantages of the slow growth rate. Meanwhile, a more extended raising period could enhance the performance of the EYR.

#### 3.2.2. ELR Trend

**Figure 4:**
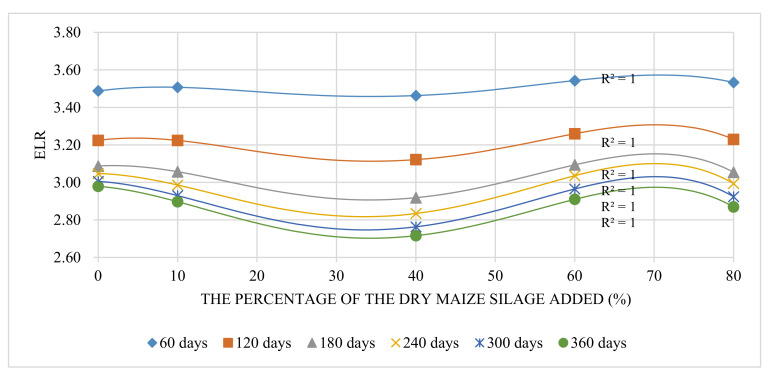
The trend of ELR change with different percentages of maize silage added.

ELR reflects the environmental pressure caused by a system. The linear fitting result (*R*^2^) between each period and its maximum EYR value was 0.99. It has been recognized that the general trend of maximum EYR value increases with the breeding period increased. This means that a longer breeding period can bring higher EYR value. In this trial, 360 days is the upper limit of the ecological farming system. Therefore, the optimal breeding period is 360 days. The fitting curve of ELR is a quartic equation (Figure 4). Overall, the ELR decreased with the increased raising period, and ELR yielded a “decrease-increase-decrease” trend with the increased percentage of maize silage. The ELR curve sunk in the range of 30–40% of maize silage added, but the gaps of ELR from 0% to 80% were heavily limited, i.e., the maximum values were only 3.2–9.2% higher than the minimum values. The extreme ELR values of different raising periods above 180 days were similar, with a change range of only 3.1–7.0%. The result indicates that a too-short raising period (<180 days) might cause high environmental pressure; pigs raised with 34.3% maize silage in 360 days would undergo the slightest environmental pressure.

#### 3.2.3. ESI Trend

**Figure 5:**
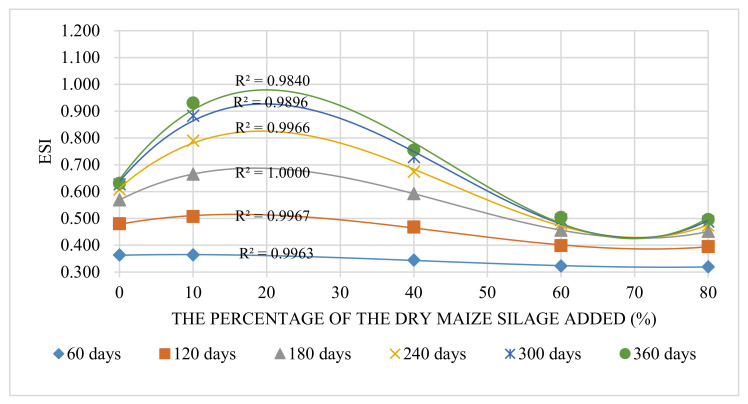
The trend of ESI change with different percentages of maize silage added.

The fitting curves of ESI were similar to those of EYR (Figure 5). The linear fitting result (*R*^2^) between each period and its maximum ESI value was 0.87. It can be believed that the general trend of maximum ESI value increases with the breeding period increased. In this trial, 360 days is the upper limit of the ecological farming system. Therefore, the optimal breeding period is 360 days. These curves were cubic equations with maximum values in the range of 10–20% and minimum values in the range of 60–80%. The linear relationship between ESI and maize silage percentage decreased with the increased raising period. ESI performed best in the range of 10–30% maize silage added when the raising period was more than 180 days. The results above show that the advantages of added silage could not eliminate the disadvantages of the slow growth rate when 40–80% maize silage was added. The best ESI performance existed below the range of 20% maize silage added when the raising period was less than 180 days. This means that a too-short raising period was not suitable for developing EPRSs with maize silage added. Overall, the longer the feeding period, the better the ESI performed. In this section, the best point was 19.9% silage maize added with a 360-day raising period.

#### 3.2.4. UEV Trend

**Figure 6:**
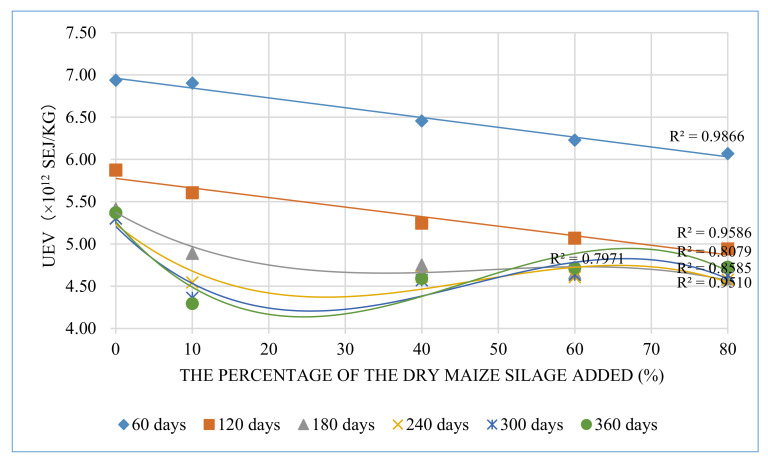
The trend of UEV changes with different percentages of maize silage.

The trend of UEV decreased with the increased maize silage percentage when the raising period was below 120 days (Figure 6), but when the raising period was over 120 days, the trend first decreased and then increased. The linear fitting result (*R*^2^) between each period and its maximum UEV value was 0.81. It shows that the general trend of maximum UEV value increases with the increase of the breeding period. In this trial, 360 days is the upper limit of the ecological farming system. Therefore, the optimal breeding period is 360 days. The linear relationship between UEV and maize silage percentage decreased with the increased raising period. Generally, UEV performed better in the range of 10–30% maize silage added than with other concentrations when the raising period was below 180 days. When the raising period (>180 days) increased, the advantages of the low UEV of maize silage were eliminated by the increased amount of feed consumed and facility wear. For ESI, the best point was 24.6% maize silage added in 360 days.

### 3.3. Economic Analysis

**Figure 7:**
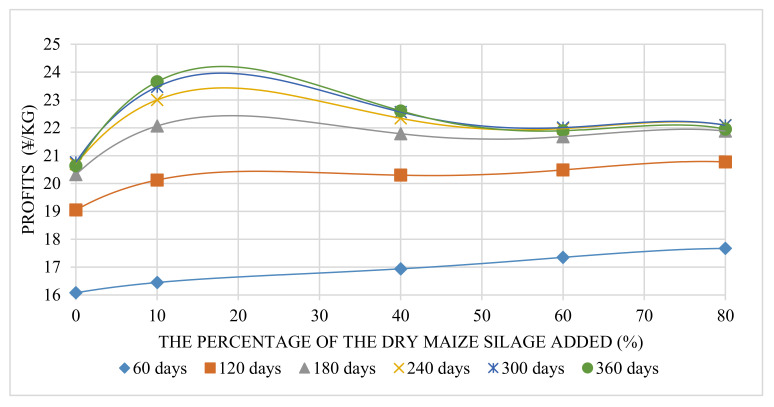
Profits were gained with different percentages of sun-dried maize silage added.

**Figure 8:**
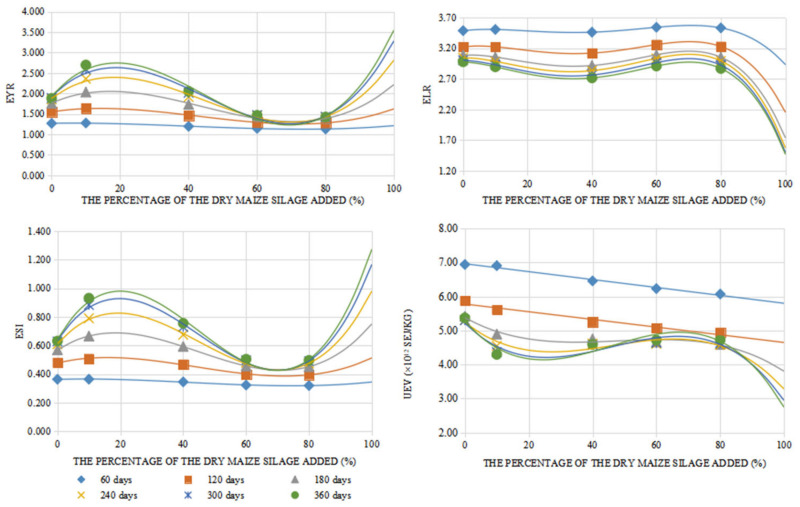
Predicted change trends of main indices with different percentages of maize silage.

Economic profit increased with the changed raising period (Table 6), but the increasing rate of profit dropped with an increased period. Meanwhile, feed adding more maize silage did not gain more economic profits for the same raising period (Figure 7). The matched curves regarding the percentage of maize silage and profit were quartic equations with two extreme points. With the increased raising period, the maximum point changed from the second extreme point (60–80%) to the first extreme point (10–30%). The linear fitting result (*R*^2^) between each period and its maximum economic profit value was 0.85. It has been recognized that the general trend of maximum economic profit value increases with the breeding period increased. This means that a longer breeding period can bring higher financial profit. In this trial, 360 days is the upper limit of the ecological farming system. Therefore, the optimal breeding period is 360 days. As Figure 8 shows, the maximum profit was located on the 360-day curve whose maximum point was 24.20 ¥/kg (18.0%). The above result means that feed with about 18.0% maize silage added with a 360-day raising period could gain the most economic benefits. This point represents better pig live bodyweight performance and better cost control. Feed without any maize silage added showed inadequate cost control.

### 3.4. Balance Point of EPRSs

Under the emergy evaluation, the points with the best performance of EYR, ELR, UEV, and ESI were, respectively, at 19.0%, 34.3%, 24.6%, and 19.9% of maize silage added with 360 days; 18.0% maize silage added to the feed with 360 days obtained best economic profit. Based on the median principle, the median of these values would affect the entire performance least. At this point, the performance of EYR, ELR, UEV, ESI, and economic profit were only 0.04%, 3.0%, 0.8%, 0.0%, and 0.1% lower than their maximum values, respectively. Generally, 0% maize silage added feed was widely used in ecological pig raising systems to gain a faster growth rate. The performances of EYR, ELR, UEV, ESI, and economic profit from the balance point were 41.3%, 6.5%, 20.8%, 52.0%, and 17.2%, respectively, better than that of the 0% maize silage added raising system. These results show that the balance point could account for environmental pressure and economic profits. In the actual pig raising process, 19.9% sun-dried maize silage addition would be too accurate for the managers to prepare the feed. Therefore, the 20% sun-dried maize silage added to the feed is suitable.

## 4. Discussion

### 4.1. Predictions of Feed with More Than 80% Maize Silage Added

**Figure 9:**
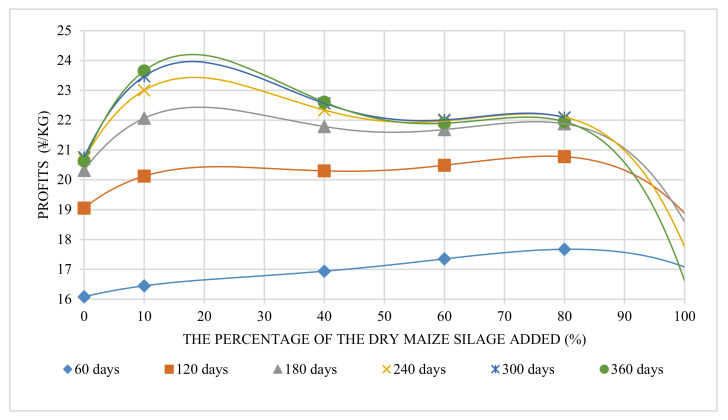
Predicted trends of profits gained with different percentages of sun-dried maize silage added.

Before the 1980s, pigs were mainly raised feeding boiled grass with limited grain in China. Such feed can be regarded as nearly 100% crude fiber added. As a result, the pig raising period could be one or two years. With the development of the economy, more and more Chinese recall the taste of the pork eaten in their childhood. They consider the pigs raised longer with more roughage would gain better taste. Demand determines the market. Many farms have started to raise pigs under the Chinese traditional method. This section will simulate and achieve the performance of such raising method with the models we obtained in Section 2.4. In this part, we assume that such action would not affect the growth model’s equations when soybean meal is replaced by maize silage. All the results here reflect some trends of these changed indices, but the degree of the change range will not be accurate. When extending the upper limit to 100% with the previous trends (Figure 9), it was found that all emergy indices showed better performance with the maize silage percentage increasing in the 80–100% range. When the percentage was above 95%, the performance exceeded that when 19.9% maize silage was added. As the prediction showed, all emergy indices performed best when the EPRS added 100% maize silage to the feed with 360 days. However, the economic profit curves sharply decreased when the percentage of maize silage changed from 80% to 100%. When 100% maize silage was added to the feed, the economic profit was only 69.0% of the best profit. This shows that the Chinese traditional pig raising system could result in good environmental performance but would sacrifice some economic profit. The longer period corresponded to fewer profits.

### 4.2. Performance of Feed with Maize Silage Added in Pig Raising Systems

As a forage, the yield of silage maize is remarkably higher than common maize; the silage maize yield (dry weight) can even be three times higher than that of common maize [12]. That means the UEV and the cost of maize silage could be much less than that of maize grain. At first, we predicted that environmental pressure and system sustainability would keep increasing with the increased maize silage percentage. However, the results show that the performance concerning the environment and sustainability changed with the increased maize silage percentage. Consequently, feed with a suitable portion of maize silage added should be good for both economic profit and environmental impact. In this study, ecological pigs fed with 19.9% maize silage added had the most balanced performance.

The equations of the pig growth models showed that the maximum final weight was with pigs fed with about 10% maize silage added to the feed. Under the calculation of the pig growth models, the final live body weight of group B could reach 370.7 kg. However, under the calculation of the pig growth models*,* the final live body weights of groups A, C, D, and E were only 188.8 kg, 224.6 kg, 153.8 kg, and 148.6 kg, respectively. There was no doubt that more than 40% sun-dried maize silage added to the feed would affect the final weight significantly. The low percentage of maize silage added to the feed could increase the richness of bacterial species and promote digestion and absorption, decreasing the occurrence of gastrointestinal diseases [13,49]. However, 40–80% of maize silage added to the feed would decrease the feed retention time in the intestines and affect the absorption of nutrients from soybean or maize [16,50]. There was no doubt that the final weight would be lower. If the percentages of maize silage were high enough (100%), such a raising system would tend to be like the Chinese conventional pig raising system before the 1980s—a much longer raising period was definitively needed. Section 4.1 shows the actual change trend of the indices, in which a high percentage of maize silage added (>95%) reduced the total emergy invested and did not affect the final weight more significantly than the 40–80% range did. It is easy to understand why such a high percentage of maize silage could gain excellent performance in terms of environmental impact.

### 4.3. Policy of Forage Planting

China’s Ministry of Agriculture recently encouraged farmers to plant forage crops instead of grain crops. Such a policy is good not only to meet the feed demand of pig husbandry, but also to reduce the application of chemical fertilizer and avoid the degradation of cultivated land. Meanwhile, it can also reduce the extent of pigs fed maize grain to compete with humans for crops. The tillage method of maize silage could increase the utilization capacity of biomass and reduce the energy invested in treating the byproducts of crops (straw crushing and burying). Moreover, straw returned to the field might cause plant diseases and pests and increase the greenhouse emissions of the field [51,52,53]. Therefore, changing the harvest method from maize grain to maize silage could decrease the amount of chemical pesticides consumed and the global warming potential. Such change increases the planting system’s sustainability and enhances the sustainability of the pig raising system.

### 4.4. Guidelines for Farmers

Farmers operating ecological farms that combine planting and breeding could change some maize grain planting fields to maize silage fields. The planting methods of silage maize are similar to the high close maize planting method, and 20% maize silage (dry weight) could be added to the feed of ecological pigs to replace some maize grain. This range of added maize silage could result in good economic profit and good environmental impact. Under such a raising method, the feed conversion ratio would be around 4.5, which is acceptable for ecological raising systems. The raising period should be controlled to nearly a year in order to neutralize the time delay caused by plant fiber added to the feed. A long enough raising time could ensure that the raising system would not miss the fast growth rate period. Of course, farmers could add more than 95% maize silage to the feed for the best environmental impact, but with some economic losses.

## 5. Conclusions

With the development of green, ecological, and organic food, it is common to add plant fiber materials to the feed during the pig raising period. However, before our trials, the precision feed concentration that is good for economic profit and the environment was unclear. This study found that 19.9% maize silage added with a 360-day raising period was the best balance point between environmental impact and economic profit. At this point, the performance of EYR, ELR, UEV, ESI, and the economic profit were only 0.04%, 3.0%, 0.8%, 0.0%, and 0.1%, respectively, lower than their corresponding maximum values. However, the performances of the balance point were much better than with traditional ecological feeding (0% silage added). Additionally, such degree of integrated performance decline was acceptable. In the actual raising process, 20% sun-dried maize silage added is easy to implement, therefore we recommend the addition of 20% of sun-dried maize silage in ecological pig-raising system during the fattening period.

## Figures and Tables

**Table 1:** Composition of feed (by mass) of ecological pig-raising systems (EPRSs).

Component	Units	A	B	C	D	E
Sun-dried maize silage	%	0	10	40	60	80
Maize grain	%	75	70	35	20	0
Wheat bran meal	%	10	5	10	5	5
Soya bean meal	%	15	15	15	15	15

**Table 2:** Raw data of weight (Wt) and cumulative feed consumption (CFI).

Group	Raising Period (Days)	W_t_ ^1^ (kg)	CFI ^2^(kg)
A	0	36.19 ± 6.45	0.00
	32	48.88 ± 8.17	65.58
	62	67.19 ± 12.93	138.18
	138	114.67 ± 15.96	349.37
B	0	29.06 ± 5.10	0.00
	37	43.10 ± 6.99	60.40
	78	64.72 ± 12.04	177.56
	122	96.87 ± 17.90	322.73
C	0	34.13 ± 10.24	0.00
	32	44.06 ± 13.10	47.55
	62	62.81 ± 17.47	116.29
	138	112.00 ± 12.94	350.04
D	0	24.50 ± 1.17	0.00
	37	42.21 ± 3.32	50.91
	78	58.78 ± 3.84	122.80
	122	85.33 ± 8.37	223.06
E	0	23.00 ± 3.35	0.00
	37	39.96 ± 3.86	49.28
	78	57.57 ± 4.51	119.42
	122	84.10 ± 7.04	234.25

Notes: ^1^ W_t_ is live pig weight, ^2^ CFI is the cumulative feed intake. A, B, C, D, E, F were the groups added 0%, 10%, 40%, 60% and 80% sun-dried silage maize, respectively.

**Table 3:** Details of growth and CFI models.

Group	Growth Model	*R* ^2^	CFI Model	*R* ^2^
A	W_t_ = 188.79/(1 + 4.31 × exp(−0.014 × t))	0.9994	CFI = 5.75 × Wt^0.95 − 171.45	0.9993
B	W_t_ = 370.75/(1 + 11.73 × exp(−0.012 × t))	0.9999	CFI = 6.96 × Wt^0.93 − 161.83	0.9986
C	W*_t_* = 224.63/(1 + 5.81 × exp(−0.013 × t))	0.9981	CFI = 1.24 × Wt^1.25 − 98.27	0.9992
D	W*_t_* = 153.83/(1 + 4.95 × exp(−0.015 × t))	0.9953	CFI = 1.10 × Wt^1.25 − 62.06	0.9977
E	W*_t_* = 148.62/(1 + 5.16 × exp(−0.016 × t))	0.9970	CFI = 0.47 × Wt^1.44 − 43.96	0.9997

Notes: Wt is live pig weight, CFI is the cumulative feed intake. A, B, C, D, E, F were the groups added 0%, 10%, 40%, 60% and 80% sun-dried silage maize, respectively.

**Table 4:** Details of Wt and CFI.

Raising Period (Days)	Item	Unit	A	B	C	D	E
60	Final weight	kg	71.78	72.59	71.27	70.95	71.86
	Total feed consumed	kg	142.11	157.93	130.91	116.28	126.57
120	Final weight	kg	110.12	121.93	112.14	103.97	104.68
	Total feed consumed	kg	309.38	387.86	324.78	255.49	286.22
180	Final weight	kg	143.78	184.11	153.06	128.53	127.58
	Total feed consumed	kg	453.74	668.25	537.66	366.72	411.97
240	Final weight	kg	166.01	246.57	184.43	142.33	139.58
	Total feed consumed	kg	548.16	943.05	711.02	431.68	482.04
300	Final weight	kg	178.08	296.55	203.91	148.89	144.93
	Total feed consumed	kg	599.14	1159.30	822.52	463.13	514.20
360	Final weight	kg	183.94	329.76	214.47	151.76	147.15
	Total feed consumed	kg	623.83	1301.50	884.10	477.01	527.67

Notes: A, B, C, D, E, F were the groups added 0%, 10%, 40%, 60% and 80% sun-dried silage maize, respectively.

**Table 5:** Main emergy indices of EPRSs.

Index	Units		Days	60	120	180	240	300	360
		Groups							
Emergy yield ratio (EYR)	-	A		1.267	1.547	1.757	1.853	1.886	1.879
		B		1.275	1.635	2.033	2.358	2.586	2.696
		C		1.194	1.460	1.729	1.911	2.014	2.050
		D		1.143	1.299	1.410	1.455	1.469	1.465
		E		1.128	1.275	1.379	1.418	1.428	1.423
Emergy sustainability index (ESI)	-	A		0.363	0.480	0.569	0.608	0.627	0.631
		B		0.364	0.507	0.665	0.790	0.883	0.931
		C		0.345	0.468	0.592	0.674	0.729	0.755
		D		0.323	0.399	0.456	0.479	0.496	0.504
		E		0.319	0.395	0.451	0.473	0.488	0.496
Environmental loading ratio (ELR)	-	A		3.488	3.224	3.087	3.047	3.006	2.979
		B		3.507	3.224	3.057	2.986	2.929	2.897
		C		3.463	3.121	2.919	2.834	2.763	2.716
		D		3.543	3.260	3.094	3.036	2.965	2.910
		E		3.533	3.230	3.054	2.994	2.924	2.869
Unit emergy value (UEV)	seJ/kg	A		6.94 × 10^12^	5.87 × 10^12^	5.42 × 10^12^	5.31 × 10^12^	5.30 × 10^12^	5.37 × 10^12^
		B		6.90 × 10^12^	5.60 × 10^12^	4.89 × 10^12^	4.54 × 10^12^	4.36 × 10^12^	4.29 × 10^12^
		C		6.46 × 10^12^	5.24 × 10^12^	4.75 × 10^12^	4.61 × 10^12^	4.57 × 10^12^	4.59 × 10^12^
		D		6.23 × 10^12^	5.07 × 10^12^	4.66 × 10^12^	4.61 × 10^12^	4.63 × 10^12^	4.72 × 10^12^
		E		6.07 × 10^12^	4.94 × 10^12^	4.59 × 10^12^	4.58× 10^12^	4.63 × 10^12^	4.72 × 10^12^

**Table 6:** Economic profit of EPRSs.

	Days	Units	60	120	180	240	300	360
Group								
A		¥/kg	16.08	19.05	20.32	20.70	20.76	20.63
B		¥/kg	16.45	20.13	22.06	23.00	23.47	23.65
C		¥/kg	16.94	20.30	21.79	22.34	22.57	22.61
D		¥/kg	17.35	20.49	21.68	21.97	22.01	21.89
E		¥/kg	17.67	20.78	21.88	22.10	22.09	21.95

Note: Raw data can be found in the Supplementary Materials (Tables S2 and S3).

## Data Availability

Data sharing is not applicable, as no new data were generated or analysed during this study.

## Supplementary Materials

The following supporting information can be downloaded at: https://www.mdpi.com/article/10.3390/ani12111446/s1, Table S1. Emergy inputs and outputs details of ecological livestock production systems, Table S2. Cost details of ecological livestock production systems (per pig), Table S3. Economic indices of ecological livestock production systems (per pig).
