# Supplementary material for: Precision Feeding in Ecological Pig-Raising Systems with Maize Silage
Source: Animals (Basel). 2022 Jun 3;12(11):1446. doi: 10.3390/ani12111446 (PMC9179495; doi:10.3390/ani12111446)
Supplement: Supplementary file 1 [file animals-12-01446-s001.zip › animals-1679741-supplementary.pdf]

Supplementary material A

The units of the following results were the inputs of per live pig.

Table S1. Emergy inputs and outputs details of ecological livestock production systems

**Percentage of the maize silage added**				**0%**		**10%**		**40%**		**60%**		**80%**	
Item	Unit	RNFa	UEVb	Raw data	Emergy	Raw data	Emergy	Raw data	Emergy	Raw data	Emergy	Raw data	Emergy
**Raising period**								**60 days**					
						Natural environmental inputs							
sun c	J	1.00	1.00E+00	3.25E+09	3.25E+09	2.60E+09	2.60E+09	3.25E+09	3.25E+09	2.59E+09	2.59E+09	2.59E+09	2.59E+09
wind c	J	1.00	1.86E+03	9.00E+05	1.67E+09	7.14E+05	1.33E+09	8.94E+05	1.66E+09	7.20E+05	1.34E+09	7.14E+05	1.33E+09
Rain c	J	1.00	2.35E+04	1.82E+06	4.27E+10	1.45E+06	3.41E+10	1.82E+06	4.27E+10	1.45E+06	3.41E+10	1.45E+06	3.41E+10
Ground water	J	0.00	1.86E+05	1.60E+06	2.97E+11	1.91E+06	3.55E+11	1.60E+06	2.96E+11	1.92E+06	3.56E+11	1.91E+06	3.55E+11
						Local materials							
Silage maize(dry weight)	kg	0.24	1.65E+11	0.00E+00	0.00E+00	1.58E+01	2.61E+12	5.24E+01	8.64E+12	6.98E+01	1.15E+13	1.01E+02	1.67E+13
Maize	kg	0.24	5.87E+11	1.07E+02	6.26E+13	1.11E+02	6.49E+13	4.58E+01	2.69E+13	2.33E+01	1.37E+13	0.00E+00	0.00E+00
Soybean meal	kg	0.33	1.42E+12	2.13E+01	3.04E+13	2.37E+01	3.37E+13	1.96E+01	2.80E+13	1.74E+01	2.48E+13	1.90E+01	2.70E+13
Wheat bran	kg	0.41	8.32E+11	1.42E+01	1.18E+13	7.90E+00	6.57E+12	1.31E+01	1.09E+13	5.81E+00	4.84E+12	6.33E+00	5.27E+12
						Purchased materials or services							
Piglet	Kg	0.21	9.02E+12	4.00E+01	3.61E+14	4.00E+01	3.61E+14	4.00E+01	3.61E+14	4.00E+01	3.61E+14	4.00E+01	3.61E+14
Disinfectants	g	0.00	1.27E+09	2.78E+01	3.54E+10	2.78E+01	3.54E+10	2.78E+01	3.54E+10	2.78E+01	3.54E+10	2.78E+01	3.54E+10
Vaccine	g	0.00	1.89E+10	1.05E+00	1.98E+10	9.84E-01	1.86E+10	3.58E-01	6.75E+09	9.84E-01	1.86E+10	9.84E-01	1.86E+10
Micro-biological additives	¥	0.20	3.11E+11	1.25E+01	3.88E+12	1.25E+01	3.88E+12	1.25E+01	3.88E+12	1.25E+01	3.88E+12	1.25E+01	3.88E+12
Electricity	J	0.09	2.17E+05	1.39E+06	3.01E+11	1.39E+06	3.01E+11	1.39E+06	3.01E+11	1.39E+06	3.01E+11	1.39E+06	3.01E+11
Steel	g	0.00	3.52E+09	7.20E+02	2.54E+12	7.20E+02	2.54E+12	7.20E+02	2.54E+12	7.20E+02	2.54E+12	7.20E+02	2.54E+12
Concrete	g	0.00	4.42E+08	5.13E+03	2.27E+12	5.13E+03	2.27E+12	5.13E+03	2.27E+12	5.13E+03	2.27E+12	5.13E+03	2.27E+12
Glass	g	0.00	2.90E+07	2.63E+01	7.63E+08	2.63E+01	7.63E+08	2.63E+01	7.63E+08	2.63E+01	7.63E+08	2.63E+01	7.63E+08
Building	¥	0.05	3.11E+11	1.25E+00	3.89E+11	1.25E+00	3.89E+11	1.25E+00	3.89E+11	1.25E+00	3.89E+11	1.25E+00	3.89E+11

Facilities	¥	0.05	3.11E+11	6.24E+00	1.94E+12	6.24E+00	1.94E+12	6.24E+00	1.94E+12	6.24E+00	1.94E+12	6.24E+00	1.94E+12
Medicine	¥	0.05	3.11E+11	3.50E+01	1.09E+13	3.44E+01	1.07E+13	1.07E+01	3.34E+12	1.48E+01	4.59E+12	1.48E+01	4.59E+12
Labor	J	0.60	5.73E+06	1.75E+06	1.00E+13	1.77E+06	1.01E+13	1.75E+06	1.00E+13	1.77E+06	1.01E+13	1.77E+06	1.01E+13
Outputs	body weight	kg		71.77958		72.58556		71.27186		70.94967		71.85844	
Total emergy					4.98E+14		5.01E+14		4.60E+14		4.42E+14		4.36E+14
**Raising period**									**120 days**				
Natural environmental inputs													
sun c	J	1.00	1.00E+00	6.49E+09	6.49E+09	5.20E+09	5.20E+09	6.49E+09	6.49E+09	5.18E+09	5.18E+09	5.18E+09	5.18E+09
wind c	J	1.00	1.86E+03	1.80E+06	3.34E+09	1.43E+06	2.65E+09	1.79E+06	3.32E+09	1.44E+06	2.67E+09	1.43E+06	2.65E+09
Rain c	J	1.00	2.35E+04	3.64E+06	8.54E+10	2.90E+06	6.82E+10	3.64E+06	8.54E+10	2.90E+06	6.82E+10	2.90E+06	6.82E+10
Ground water	J	0.00	1.86E+05	3.20E+06	5.95E+11	3.83E+06	7.11E+11	3.19E+06	5.92E+11	3.84E+06	7.13E+11	3.83E+06	7.11E+11
Local materials													
Silage maize(dry weight)	kg	0.24	1.65E+11	0	0.00E+00	38.78611	6.40E+12	129.9134	2.14E+13	153.2924	2.53E+13	228.9723	3.78E+13
Maize	kg	0.24	5.87E+11	232.0325	1.36E+14	271.5028	1.59E+14	113.6742	6.67E+13	51.09747	3.00E+13	0	0.00E+00
Soybean meal	kg	0.33	1.42E+12	46.4065	6.61E+13	58.17916	8.29E+13	48.71753	6.94E+13	38.3231	5.46E+13	42.93231	6.11E+13
Wheat bran	kg	0.41	8.32E+11	30.93767	2.57E+13	19.39305	1.61E+13	32.47835	2.70E+13	12.77437	1.06E+13	14.31077	1.19E+13
Purchased materials or services													
Piglet	Kg	0.21	9.02E+12	4.00E+01	3.61E+14	4.00E+01	3.61E+14	4.00E+01	3.61E+14	4.00E+01	3.61E+14	4.00E+01	3.61E+14
Disinfectants	g	0.00	1.27E+09	2.78E+01	3.54E+10	2.78E+01	3.54E+10	2.78E+01	3.54E+10	2.78E+01	3.54E+10	2.78E+01	3.54E+10
Vaccine	g	0.00	1.89E+10	2.10E+00	3.96E+10	1.97E+00	3.71E+10	7.15E-01	1.35E+10	1.97E+00	3.71E+10	1.97E+00	3.71E+10
Micro-biological additives	¥	0.20	3.11E+11	2.50E+01	7.76E+12	2.50E+01	7.76E+12	2.50E+01	7.76E+12	2.50E+01	7.76E+12	2.50E+01	7.76E+12
Electricity	J	0.09	2.17E+05	2.77E+06	6.03E+11	2.77E+06	6.03E+11	2.77E+06	6.03E+11	2.77E+06	6.03E+11	2.77E+06	6.03E+11
Steel	g	0.00	3.52E+09	7.20E+02	2.54E+12	7.20E+02	2.54E+12	7.20E+02	2.54E+12	7.20E+02	2.54E+12	7.20E+02	2.54E+12
Concrete	g	0.00	4.42E+08	5.13E+03	2.27E+12	5.13E+03	2.27E+12	5.13E+03	2.27E+12	5.13E+03	2.27E+12	5.13E+03	2.27E+12
Glass	g	0.00	2.90E+07	2.63E+01	7.63E+08	2.63E+01	7.63E+08	2.63E+01	7.63E+08	2.63E+01	7.63E+08	2.63E+01	7.63E+08

Building	¥	0.05	3.11E+11	1.25E+00	3.89E+11	1.25E+00	3.89E+11	1.25E+00	3.89E+11	1.25E+00	3.89E+11	1.25E+00	3.89E+11
Facilities	¥	0.05	3.11E+11	6.24E+00	1.94E+12	6.24E+00	1.94E+12	6.24E+00	1.94E+12	6.24E+00	1.94E+12	6.24E+00	1.94E+12
Medicine	¥	0.05	3.11E+11	7.00E+01	2.18E+13	6.89E+01	2.14E+13	2.15E+01	6.68E+12	2.95E+01	9.18E+12	2.95E+01	9.18E+12
Labor	J	0.60	5.73E+06	3.50E+06	2.01E+13	3.54E+06	2.03E+13	3.50E+06	2.01E+13	3.54E+06	2.03E+13	3.54E+06	2.03E+13
Outputs	body weight	kg		110.1206		121.9301		112.142		103.9724		104.6843	
Total emergy					6.47E+14		6.83E+14		5.88E+14		5.27E+14		5.17E+14
**Raising period**								**180 days**					
Natural environmental inputs													
sun c	J	1.00	1.00E+00	9.74E+09	9.74E+09	7.79E+09	7.79E+09	9.74E+09	9.74E+09	7.78E+09	7.78E+09	7.78E+09	7.78E+09
wind c	J	1.00	1.86E+03	2.70E+06	5.01E+09	2.14E+06	3.98E+09	2.68E+06	4.98E+09	2.16E+06	4.01E+09	2.14E+06	3.98E+09
Rain c	J	1.00	2.35E+04	5.45E+06	1.28E+11	4.36E+06	1.02E+11	5.45E+06	1.28E+11	4.36E+06	1.02E+11	4.36E+06	1.02E+11
Ground water	J	0.00	1.86E+05	4.81E+06	8.92E+11	5.74E+06	1.07E+12	4.79E+06	8.89E+11	5.76E+06	1.07E+12	5.74E+06	1.07E+12
Local materials													
Silage maize(dry weight)	kg	0.24	1.65E+11	0.00E+00	0.00E+00	6.68E+01	1.10E+13	2.15E+02	3.55E+13	2.20E+02	3.63E+13	3.30E+02	5.44E+13
Maize	kg	0.24	5.87E+11	3.40E+02	2.00E+14	4.68E+02	2.75E+14	1.88E+02	1.10E+14	7.33E+01	4.31E+13	0.00E+00	0.00E+00
Soybean meal	kg	0.33	1.42E+12	6.81E+01	9.69E+13	1.00E+02	1.43E+14	8.06E+01	1.15E+14	5.50E+01	7.83E+13	6.18E+01	8.80E+13
Wheat bran	kg	0.41	8.32E+11	4.54E+01	3.78E+13	3.34E+01	2.78E+13	5.38E+01	4.47E+13	1.83E+01	1.53E+13	2.06E+01	1.71E+13
Purchased materials or services													
Piglet	Kg	0.21	9.02E+12	4.00E+01	3.61E+14	4.00E+01	3.61E+14	4.00E+01	3.61E+14	4.00E+01	3.61E+14	4.00E+01	3.61E+14
Disinfectants	g	0.00	1.27E+09	2.78E+01	3.54E+10	2.78E+01	3.54E+10	2.78E+01	3.54E+10	2.78E+01	3.54E+10	2.78E+01	3.54E+10
Vaccine	g	0.00	1.89E+10	3.15E+00	5.94E+10	2.95E+00	5.57E+10	1.07E+00	2.02E+10	2.95E+00	5.57E+10	2.95E+00	5.57E+10
Micro-biological additives	¥	0.20	3.11E+11	3.74E+01	1.16E+13	3.74E+01	1.16E+13	3.74E+01	1.16E+13	3.74E+01	1.16E+13	3.74E+01	1.16E+13
Electricity	J	0.09	2.17E+05	4.16E+06	9.04E+11	4.16E+06	9.04E+11	4.16E+06	9.04E+11	4.16E+06	9.04E+11	4.16E+06	9.04E+11
Steel	g	0.00	3.52E+09	7.20E+02	2.54E+12	7.20E+02	2.54E+12	7.20E+02	2.54E+12	7.20E+02	2.54E+12	7.20E+02	2.54E+12

Concrete	g	0.00	4.42E+08	5.13E+03	2.27E+12	5.13E+03	2.27E+12	5.13E+03	2.27E+12	5.13E+03	2.27E+12	5.13E+03	2.27E+12
Glass	g	0.00	2.90E+07	2.63E+01	7.63E+08	2.63E+01	7.63E+08	2.63E+01	7.63E+08	2.63E+01	7.63E+08	2.63E+01	7.63E+08
Building	¥	0.05	3.11E+11	1.25E+00	3.89E+11	1.25E+00	3.89E+11	1.25E+00	3.89E+11	1.25E+00	3.89E+11	1.25E+00	3.89E+11
Facilities	¥	0.05	3.11E+11	6.24E+00	1.94E+12	6.24E+00	1.94E+12	6.24E+00	1.94E+12	6.24E+00	1.94E+12	6.24E+00	1.94E+12
Medicine	¥	0.05	3.11E+11	1.05E+02	3.26E+13	1.03E+02	3.21E+13	3.22E+01	1.00E+13	4.43E+01	1.38E+13	4.43E+01	1.38E+13
Labor	J	0.60	5.73E+06	5.26E+06	3.01E+13	5.31E+06	3.04E+13	5.26E+06	3.01E+13	5.31E+06	3.04E+13	5.31E+06	3.04E+13
Outputs	body weight	kg		1.44E+02		1.84E+02		1.53E+02		1.29E+02		1.28E+02	
Total emergy					7.79E+14		9.00E+14		7.27E+14		5.99E+14		5.85E+14
**Raising period**								**240 days**					
Natural environmental inputs													
sun c	J	1.00	1.00E+00	1.30E+10	1.30E+10	1.04E+10	1.04E+10	1.30E+10	1.30E+10	1.04E+10	1.04E+10	1.04E+10	1.04E+10
wind c	J	1.00	1.86E+03	3.60E+06	6.68E+09	2.86E+06	5.30E+09	3.58E+06	6.64E+09	2.88E+06	5.35E+09	2.86E+06	5.30E+09
Rain c	J	1.00	2.35E+04	7.27E+06	1.71E+11	5.81E+06	1.36E+11	7.27E+06	1.71E+11	5.81E+06	1.36E+11	5.81E+06	1.36E+11
Ground water	J	0.00	1.86E+05	6.41E+06	1.19E+12	7.66E+06	1.42E+12	6.38E+06	1.18E+12	7.68E+06	1.43E+12	7.66E+06	1.42E+12
Local materials													
Silage maize(dry weight)	kg	0.24	1.65E+11	0	0.00E+00	94.30547	1.56E+13	284.4084	4.69E+13	259.0082	4.27E+13	385.6342	6.36E+13
Maize	kg	0.24	5.87E+11	411.1193	2.41E+14	660.1383	3.88E+14	248.8573	1.46E+14	86.33605	5.07E+13	0	0.00E+00
Soybean meal	kg	0.33	1.42E+12	82.22386	1.17E+14	141.4582	2.01E+14	106.6531	1.52E+14	64.75204	9.22E+13	72.30642	1.03E+14
Wheat bran	kg	0.41	8.32E+11	54.81591	4.56E+13	47.15274	3.92E+13	71.1021	5.92E+13	21.58401	1.80E+13	24.10214	2.01E+13
Purchased materials or services													
Piglet	Kg	0.21	9.02E+12	4.00E+01	3.61E+14	4.00E+01	3.61E+14	4.00E+01	3.61E+14	4.00E+01	3.61E+14	4.00E+01	3.61E+14
Disinfectants	g	0.00	1.27E+09	2.78E+01	3.54E+10	2.78E+01	3.54E+10	2.78E+01	3.54E+10	2.78E+01	3.54E+10	2.78E+01	3.54E+10
Vaccine	g	0.00	1.89E+10	4.20E+00	7.92E+10	3.94E+00	7.42E+10	1.43E+00	2.70E+10	3.94E+00	7.42E+10	3.94E+00	7.42E+10
Micro-biological additives	¥	0.20	3.11E+11	4.99E+01	1.55E+13	4.99E+01	1.55E+13	4.99E+01	1.55E+13	4.99E+01	1.55E+13	4.99E+01	1.55E+13
Electricity	J	0.09	2.17E+05	5.54E+06	1.21E+12	5.54E+06	1.21E+12	5.54E+06	1.21E+12	5.54E+06	1.21E+12	5.54E+06	1.21E+12

Steel	g	0.00	3.52E+09	1.44E+03	5.07E+12	1.44E+03	5.07E+12	1.44E+03	5.07E+12	1.44E+03	5.07E+12	1.44E+03	5.07E+12
Concrete	g	0.00	4.42E+08	1.03E+04	4.53E+12	1.03E+04	4.53E+12	1.03E+04	4.53E+12	1.03E+04	4.53E+12	1.03E+04	4.53E+12
Glass	g	0.00	2.90E+07	5.26E+01	1.53E+09	5.26E+01	1.53E+09	5.26E+01	1.53E+09	5.26E+01	1.53E+09	5.26E+01	1.53E+09
Building	¥	0.05	3.11E+11	2.50E+00	7.77E+11	2.50E+00	7.77E+11	2.50E+00	7.77E+11	2.50E+00	7.77E+11	2.50E+00	7.77E+11
Facilities	¥	0.05	3.11E+11	1.25E+01	3.88E+12	1.25E+01	3.88E+12	1.25E+01	3.88E+12	1.25E+01	3.88E+12	1.25E+01	3.88E+12
Medicine	¥	0.05	3.11E+11	1.40E+02	4.35E+13	1.38E+02	4.28E+13	4.30E+01	1.34E+13	5.90E+01	1.84E+13	5.90E+01	1.84E+13
Labor	J	0.60	5.73E+06	7.01E+06	4.01E+13	7.08E+06	4.05E+13	7.01E+06	4.01E+13	7.08E+06	4.05E+13	7.08E+06	4.05E+13
Outputs	body weight	kg		1.66E+02		2.47E+02		1.84E+02		1.42E+02		1.40E+02	
Total emergy					8.81E+14		1.12E+15		8.51E+14		6.56E+14		6.39E+14
**Raising period**								**300 days**					
						Natural environmental inputs							
sun c	J	1.00	1.00E+00	1.62E+10	1.62E+10	1.30E+10	1.30E+10	1.62E+10	1.62E+10	1.30E+10	1.30E+10	1.30E+10	1.30E+10
wind c	J	1.00	1.86E+03	4.50E+06	8.35E+09	3.57E+06	6.63E+09	4.47E+06	8.30E+09	3.60E+06	6.68E+09	3.57E+06	6.63E+09
Rain c	J	1.00	2.35E+04	9.09E+06	2.13E+11	7.26E+06	1.71E+11	9.09E+06	2.13E+11	7.26E+06	1.71E+11	7.26E+06	1.71E+11
Ground water	J	0.00	1.86E+05	8.01E+06	1.49E+12	9.57E+06	1.78E+12	7.98E+06	1.48E+12	9.60E+06	1.78E+12	9.57E+06	1.78E+12
						Local materials							
Silage maize(dry weight)	kg	0.24	1.65E+11	0.00E+00	0.00E+00	1.16E+02	1.91E+13	3.29E+02	5.43E+13	2.78E+02	4.59E+13	4.11E+02	6.79E+13
Maize	kg	0.24	5.87E+11	4.49E+02	2.64E+14	8.12E+02	4.76E+14	2.88E+02	1.69E+14	9.26E+01	5.44E+13	0.00E+00	0.00E+00
Soybean meal	kg	0.33	1.42E+12	8.99E+01	1.28E+14	1.74E+02	2.48E+14	1.23E+02	1.76E+14	6.95E+01	9.89E+13	7.71E+01	1.10E+14
Wheat bran	kg	0.41	8.32E+11	5.99E+01	4.98E+13	5.80E+01	4.82E+13	8.23E+01	6.84E+13	2.32E+01	1.93E+13	2.57E+01	2.14E+13
						Purchased materials or services							
Piglet	Kg	0.21	9.02E+12	4.00E+01	3.61E+14	4.00E+01	3.61E+14	4.00E+01	3.61E+14	4.00E+01	3.61E+14	4.00E+01	3.61E+14
Disinfectants	g	0.00	1.27E+09	2.78E+01	3.54E+10	2.78E+01	3.54E+10	2.78E+01	3.54E+10	2.78E+01	3.54E+10	2.78E+01	3.54E+10
Vaccine	g	0.00	1.89E+10	5.25E+00	9.90E+10	4.92E+00	9.28E+10	1.79E+00	3.37E+10	4.92E+00	9.28E+10	4.92E+00	9.28E+10
Micro-biological additives	¥	0.20	3.11E+11	6.24E+01	1.94E+13	6.24E+01	1.94E+13	6.24E+01	1.94E+13	6.24E+01	1.94E+13	6.24E+01	1.94E+13

Electricity	J	0.09	2.17E+05	6.93E+06	1.51E+12	6.93E+06	1.51E+12	6.93E+06	1.51E+12	6.93E+06	1.51E+12	6.93E+06	1.51E+12
Steel	g	0.00	3.52E+09	1.44E+03	5.07E+12	1.44E+03	5.07E+12	1.44E+03	5.07E+12	1.44E+03	5.07E+12	1.44E+03	5.07E+12
Concrete	g	0.00	4.42E+08	1.03E+04	4.53E+12	1.03E+04	4.53E+12	1.03E+04	4.53E+12	1.03E+04	4.53E+12	1.03E+04	4.53E+12
Glass	g	0.00	2.90E+07	5.26E+01	1.53E+09	5.26E+01	1.53E+09	5.26E+01	1.53E+09	5.26E+01	1.53E+09	5.26E+01	1.53E+09
Building	¥	0.05	3.11E+11	2.50E+00	7.77E+11	2.50E+00	7.77E+11	2.50E+00	7.77E+11	2.50E+00	7.77E+11	2.50E+00	7.77E+11
Facilities	¥	0.05	3.11E+11	1.25E+01	3.88E+12	1.25E+01	3.88E+12	1.25E+01	3.88E+12	1.25E+01	3.88E+12	1.25E+01	3.88E+12
Medicine	¥	0.05	3.11E+11	1.75E+02	5.44E+13	1.72E+02	5.36E+13	5.37E+01	1.67E+13	7.38E+01	2.30E+13	7.38E+01	2.30E+13
Labor	J	0.60	5.73E+06	8.76E+06	5.02E+13	8.85E+06	5.07E+13	8.76E+06	5.02E+13	8.85E+06	5.07E+13	8.85E+06	5.07E+13
Outputs	body weight	kg		1.78E+02		2.97E+02		2.04E+02		1.49E+02		1.45E+02	
Total emergy					9.44E+14		1.29E+15		9.32E+14		6.90E+14		6.71E+14
**Raising period**									**360 days**				
Natural environmental inputs													
sun c	J	1.00	1.00E+00	1.95E+10	1.95E+10	1.56E+10	1.56E+10	1.95E+10	1.95E+10	1.56E+10	1.56E+10	1.56E+10	1.56E+10
wind c	J	1.00	1.86E+03	5.40E+06	1.00E+10	4.28E+06	7.95E+09	5.36E+06	9.96E+09	4.32E+06	8.02E+09	4.28E+06	7.95E+09
Rain c	J	1.00	2.35E+04	1.09E+07	2.56E+11	8.71E+06	2.05E+11	1.09E+07	2.56E+11	8.71E+06	2.05E+11	8.71E+06	2.05E+11
Ground water	J	0.00	1.86E+05	9.61E+06	1.78E+12	1.15E+07	2.13E+12	9.58E+06	1.78E+12	1.15E+07	2.14E+12	1.15E+07	2.13E+12
Local materials													
Silage maize(dry weight)	kg	0.24	1.65E+11	0	0.00E+00	130.1504	2.15E+13	353.6409	5.84E+13	286.2076	4.72E+13	422.1369	6.97E+13
Maize	kg	0.24	5.87E+11	467.8725	2.75E+14	911.0528	5.35E+14	309.4358	1.82E+14	95.40252	5.60E+13	0	0.00E+00
Soybean meal	kg	0.33	1.42E+12	93.57449	1.33E+14	195.2256	2.78E+14	132.6153	1.89E+14	71.55189	1.02E+14	79.15068	1.13E+14
Wheat bran	kg	0.41	8.32E+11	62.38299	5.19E+13	65.0752	5.41E+13	88.41022	7.36E+13	23.85063	1.98E+13	26.38356	2.20E+13
Purchased materials or services													
Piglet	Kg	0.21	9.02E+12	4.00E+01	3.61E+14	4.00E+01	3.61E+14	4.00E+01	3.61E+14	4.00E+01	3.61E+14	4.00E+01	3.61E+14
Disinfectants	g	0.00	1.27E+09	2.78E+01	3.54E+10	2.78E+01	3.54E+10	2.78E+01	3.54E+10	2.78E+01	3.54E+10	2.78E+01	3.54E+10
Vaccine	g	0.00	1.89E+10	6.30E+00	1.19E+11	5.90E+00	1.11E+11	2.15E+00	4.05E+10	5.90E+00	1.11E+11	5.90E+00	1.11E+11

Micro-biological additives	¥	0.20	3.11E+11	7.49E+01	2.33E+13	7.49E+01	2.33E+13	7.49E+01	2.33E+13	7.49E+01	2.33E+13	7.49E+01	2.33E+13
Electricity	J	0.09	2.17E+05	8.32E+06	1.81E+12	8.32E+06	1.81E+12	8.32E+06	1.81E+12	8.32E+06	1.81E+12	8.32E+06	1.81E+12
Steel	g	0.00	3.52E+09	1.44E+03	5.07E+12	1.44E+03	5.07E+12	1.44E+03	5.07E+12	1.44E+03	5.07E+12	1.44E+03	5.07E+12
Concrete	g	0.00	4.42E+08	1.03E+04	4.53E+12	1.03E+04	4.53E+12	1.03E+04	4.53E+12	1.03E+04	4.53E+12	1.03E+04	4.53E+12
Glass	g	0.00	2.90E+07	5.26E+01	1.53E+09	5.26E+01	1.53E+09	5.26E+01	1.53E+09	5.26E+01	1.53E+09	5.26E+01	1.53E+09
Building	¥	0.05	3.11E+11	2.50E+00	7.77E+11	2.50E+00	7.77E+11	2.50E+00	7.77E+11	2.50E+00	7.77E+11	2.50E+00	7.77E+11
Facilities	¥	0.05	3.11E+11	1.25E+01	3.88E+12	1.25E+01	3.88E+12	1.25E+01	3.88E+12	1.25E+01	3.88E+12	1.25E+01	3.88E+12
Medicine	¥	0.05	3.11E+11	2.10E+02	6.53E+13	2.07E+02	6.43E+13	6.44E+01	2.00E+13	8.86E+01	2.75E+13	8.86E+01	2.75E+13
Labor	J	0.60	5.73E+06	1.05E+07	6.02E+13	1.06E+07	6.08E+13	1.05E+07	6.02E+13	1.06E+07	6.08E+13	1.06E+07	6.08E+13
Outputs	body weight	kg		1.84E+02		3.30E+02		2.14E+02		1.52E+02		1.47E+02	
Total emergy					9.87E+14		1.42E+15		9.85E+14		7.16E+14		6.95E+14

[a]:The baseline used in this study is12.00E+24seJ/year standard.

[b]: Only the highest value of wind and rain was considered.

[c]: Compound fertiliser was calculated by its composition of N,P,K.

Supplementary material B

Table S2. Cost details of ecological livestock production systems (per pig)

Group	Items	Units	Raising Period (days)					
			60.00	120.00	180.00	240.00	300.00	360.00
Group A	Piglet	¥/kg	800.00	800.00	800.00	800.00	800.00	800.00
	Silage maize	¥/kg	0.00	0.00	0.00	0.00	0.00	0.00
	Maize	¥/kg	79.52	173.12	253.90	306.73	335.26	349.07
	Soybean meal	¥/kg	4.04	8.80	12.91	15.59	17.04	17.75

	Wheat bran	¥/kg	45.47	99.00	145.20	175.41	191.72	199.63
	Vaccine	¥/g	3.09	3.09	3.09	3.09	3.09	3.09
	Disinfectants	¥/g	1.58	3.15	4.73	6.30	7.88	9.45
	Micro-biological additives	¥.per pig	12.48	24.96	37.44	49.92	62.40	74.88
	Electricity	¥.J	0.21	0.42	0.63	0.84	1.05	1.26
	Steel	¥/g	3.74	3.74	3.74	7.49	7.49	7.49
	Concrete	¥/g	0.95	0.95	0.95	1.90	1.90	1.90
	Glass	¥/g	0.06	0.06	0.06	0.11	0.11	0.11
	Building	¥.per pig	1.25	1.25	1.25	2.50	2.50	2.50
	Facilities	¥.per pig	6.24	6.24	6.24	12.48	12.48	12.48
	Medicine	¥.per pig	34.98	69.96	104.94	139.92	174.90	209.88
	Labor	¥.J	5.55	11.11	16.66	22.22	27.77	33.32
Group B	Piglet	¥/kg	800.00	800.00	800.00	800.00	800.00	800.00
	Silage maize	¥/g	1.89	4.63	7.98	11.27	13.85	15.55
	Maize	¥/g	82.48	202.56	349.00	492.52	605.46	679.73
	Soybean meal	¥/g	4.49	11.03	19.01	26.83	32.98	37.03
	Wheat bran	¥/g	25.27	62.06	106.92	150.89	185.49	208.24
	Vaccine	¥/g	3.09	3.09	3.09	3.09	3.09	3.09
	Disinfectants	¥/g	1.48	2.95	4.43	5.90	7.38	8.86
	Micro-biological additives	¥.per pig	12.48	24.96	37.44	49.92	62.40	74.88
	Electricity	¥.J	0.21	0.42	0.63	0.84	1.05	1.26
	Steel	¥/g	3.74	3.74	3.74	7.49	7.49	7.49
	Concrete	¥/g	0.95	0.95	0.95	1.90	1.90	1.90

	Glass	¥/g	0.06	0.06	0.06	0.11	0.11	0.11
	Building	¥.per pig	1.25	1.25	1.25	2.50	2.50	2.50
	Facilities	¥.per pig	6.24	6.24	6.24	12.48	12.48	12.48
	Medicine	¥.per pig	34.44	68.88	103.32	137.76	172.20	206.64
	Labor	¥.J	5.61	11.22	16.83	22.44	28.05	33.67
Group C	Piglet	¥/kg	800.00	800.00	800.00	800.00	800.00	800.00
	Silage maize	¥/g	6.26	15.52	25.69	33.98	39.31	42.25
	Maize	¥/g	34.18	84.81	140.40	185.67	214.79	230.87
	Soybean meal	¥/g	3.72	9.24	15.30	20.23	23.40	25.15
	Wheat bran	¥/g	41.89	103.93	172.05	227.53	263.21	282.91
	Vaccine	¥/g	3.09	3.09	3.09	3.09	3.09	3.09
	Disinfectants	¥/g	0.54	1.07	1.61	2.15	2.68	3.22
	Micro-biological additives	¥.per pig	12.48	24.96	37.44	49.92	62.40	74.88
	Electricity	¥.J	0.21	0.42	0.63	0.84	1.05	1.26
	Steel	¥/g	3.74	3.74	3.74	7.49	7.49	7.49
	Concrete	¥/g	0.95	0.95	0.95	1.90	1.90	1.90
	Glass	¥/g	0.06	0.06	0.06	0.11	0.11	0.11
	Building	¥.per pig	1.25	1.25	1.25	2.50	2.50	2.50
	Facilities	¥.per pig	6.24	6.24	6.24	12.48	12.48	12.48
	Medicine	¥.per pig	10.74	21.48	32.22	42.96	53.70	64.44
	Labor	¥.J	5.55	11.11	16.66	22.22	27.77	33.32
Group D	Piglet	¥/kg	800.00	800.00	800.00	800.00	800.00	800.00
	Silage maize	¥/g	8.34	18.31	26.29	30.94	33.20	34.19
	Maize	¥/g	17.35	38.12	54.72	64.41	69.11	71.18

	Soybean meal	¥/g	3.31	7.27	10.43	12.28	13.18	13.57
	Wheat bran	¥/g	18.60	40.88	58.68	69.07	74.10	76.32
	Vaccine	¥/g	3.09	3.09	3.09	3.09	3.09	3.09
	Disinfectants	¥/g	1.48	2.95	4.43	5.90	7.38	8.86
	Micro-biological additives	¥.per pig	12.48	24.96	37.44	49.92	62.40	74.88
	Electricity	¥.J	0.21	0.42	0.63	0.84	1.05	1.26
	Steel	¥/g	3.74	3.74	3.74	7.49	7.49	7.49
	Concrete	¥/g	0.95	0.95	0.95	1.90	1.90	1.90
	Glass	¥/g	0.06	0.06	0.06	0.11	0.11	0.11
	Building	¥.per pig	1.25	1.25	1.25	2.50	2.50	2.50
	Facilities	¥.per pig	6.24	6.24	6.24	12.48	12.48	12.48
	Medicine	¥.per pig	14.76	29.52	44.28	59.04	73.80	88.56
	Labor	¥.J	5.61	11.22	16.83	22.44	28.05	33.67
Group E	Piglet	¥/kg	800.00	800.00	800.00	800.00	800.00	800.00
	Silage maize	¥/g	12.10	27.36	39.38	46.07	49.15	50.43
	Maize	¥/g	0.00	0.00	0.00	0.00	0.00	0.00
	Soybean meal	¥/g	3.60	8.14	11.72	13.71	14.63	15.01
	Wheat bran	¥/g	20.25	45.79	65.91	77.13	82.27	84.43
	Vaccine	¥/g	3.09	3.09	3.09	3.09	3.09	3.09
	Disinfectants	¥/g	1.48	2.95	4.43	5.90	7.38	8.86
	Micro-biological additives	¥.per pig	12.48	24.96	37.44	49.92	62.40	74.88
	Electricity	¥.J	0.21	0.42	0.63	0.84	1.05	1.26
	Steel	¥/g	3.74	3.74	3.74	7.49	7.49	7.49

	Concrete	¥/g	0.95	0.95	0.95	1.90	1.90	1.90
	Glass	¥/g	0.06	0.06	0.06	0.11	0.11	0.11
	Building	¥.per pig	1.25	1.25	1.25	2.50	2.50	2.50
	Facilities	¥.per pig	6.24	6.24	6.24	12.48	12.48	12.48
	Medicine	¥.per pig	14.76	29.52	44.28	59.04	73.80	88.56
	Labor	¥.J	5.61	11.22	16.83	22.44	28.05	33.67

Table S3. Economic indices of ecological livestock production systems (per pig)

Group	Items	Units	Raising period (days)					
			60	120	180	240	300	360
A	Price	¥	30.0	30.0	30.0	30.0	30.0	30.0
	Cost	¥	2153.4	3303.6	4313.3	4980.3	5342.4	5518.2
	Profits	¥	1154.2	2097.8	2921.6	3435.8	3696.8	3795.4
	Profits per kg live body weight	¥/kg	16.1	19.0	20.3	20.7	20.8	20.6
B	Price	¥	30.0	30.0	30.0	30.0	30.0	30.0
	Cost	¥	2177.6	3657.9	5523.2	7397.1	8896.6	9892.8
	Profits	¥	1193.9	2453.9	4062.3	5671.1	6960.1	7799.4
	Profits per kg live body weight	¥/kg	16.4	20.1	22.1	23.0	23.5	23.7
C	Price	¥	30.0	30.0	30.0	30.0	30.0	30.0
	Cost	¥	2138.2	3364.3	4591.7	5532.8	6117.2	6434.0
	Profits	¥	1207.3	2276.4	3334.4	4119.7	4601.3	4848.2
	Profits per kg live body weight	¥/kg	16.9	20.3	21.8	22.3	22.6	22.6
D	Price	¥	30.0	30.0	30.0	30.0	30.0	30.0
	Cost	¥	2128.5	3119.2	3855.9	4269.8	4466.7	4552.8
	Profits	¥	1231.0	2130.2	2786.8	3127.4	3276.8	3322.8

	Profits per kg live body weight	¥/kg	17.4	20.5	21.7	22.0	22.0	21.9
E	Price	¥	30.0	30.0	30.0	30.0	30.0	30.0
	Cost	¥	2155.8	3140.5	3827.5	4187.4	4348.0	4414.5
	Profits	¥	1269.9	2174.8	2791.5	3084.7	3201.7	3229.9
	Profits per kg live body weight	¥/kg	17.7	20.8	21.9	22.1	22.1	21.9
